# Modeling the Effect of the Metastatic Microenvironment on Phenotypes Conferred by Estrogen Receptor Mutations Using a Human Liver Microphysiological System

**DOI:** 10.1038/s41598-019-44756-5

**Published:** 2019-06-06

**Authors:** Mark T. Miedel, Dillon C. Gavlock, Shanhang Jia, Albert Gough, D. Lansing Taylor, Andrew M. Stern

**Affiliations:** 1 Drug Discovery Institute, University of Pittsburgh, Pittsburgh, PA USA; 2 Department of Computational and Systems Biology, University of Pittsburgh, Pittsburgh, PA USA; 3 School of Medicine, Tsinghua University, Beijing, China; 4 University of Pittsburgh Cancer Institute, Pittsburgh, PA USA

**Keywords:** Breast cancer, Target identification

## Abstract

Reciprocal coevolution of tumors and their microenvironments underlies disease progression, yet intrinsic limitations of patient-derived xenografts and simpler cell-based models present challenges towards a deeper understanding of these intercellular communication networks. To help overcome these barriers and complement existing models, we have developed a human microphysiological system (MPS) model of the human liver acinus, a common metastatic site, and have applied this system to estrogen receptor (ER)+ breast cancer. In addition to their hallmark constitutive (but ER-dependent) growth phenotype, different ESR1 missense mutations, prominently observed during estrogen deprivation therapy, confer distinct estrogen-enhanced growth and drug resistant phenotypes not evident under cell autonomous conditions. Under low molecular oxygen within the physiological range (~5–20%) of the normal liver acinus, the estrogen-enhanced growth phenotypes are lost, a dependency not observed in monoculture. In contrast, the constitutive growth phenotypes are invariant within this range of molecular oxygen suggesting that ESR1 mutations confer a growth advantage not only during estrogen deprivation but also at lower oxygen levels. We discuss the prospects and limitations of implementing human MPS, especially in conjunction with *in situ* single cell hyperplexed computational pathology platforms, to identify biomarkers mechanistically linked to disease progression that inform optimal therapeutic strategies for patients.

## Introduction

Despite significant advances in the detection and treatment of primary breast tumors and the development of novel therapies that increase survival, metastatic breast cancer (MBC) remains a lethal disease^1^. Approximately 14–35% of metastatic breast cancers express mutations within the ligand-binding domain (LBD) of the estrogen receptor alpha (ESR1)^2–6^. Both biochemical and clinical evidence support the hypothesis that, as a result of the constitutive signaling activity that these LBD mutations impart, ESR1 mutations are selected during estrogen deprivation therapy and drive the progression of metastatic disease^3,7,8^. Interestingly, clinical studies performing longitudinal analysis of circulating cell-free DNA (cfDNA) demonstrated that *ESR1* mutations exhibit polyclonality in individual patients and clones expressing distinct ESR1 mutations show divergent behavior with respect to drug treatment over time^8–13^. These observations suggest that distinct *ESR1* LBD mutations may confer unique, clinically relevant phenotypes. In support of this hypothesis, recent work by our group and others identified unique phenotypic differences between the two most common LBD mutations observed in the clinic, Y537S and D538G, in response to physiological levels of estrogen^14,15^.

A limitation of these studies was the exclusive use of cell autonomous conditions. In addition to differences derived from clonal heterogeneity, malignant phenotypes (i.e., immune evasion, drug resistance, metastatic potential, organ tropism, dormancy) that drive disease progression of MBC co-evolve with stromal and immune cells within the heterogeneous tumor microenvironment (TME)^16–18^. A better understanding of how cancer cells behave and function within the metastatic microenvironment is critical to the translational objective of defining the signaling networks within the TME that lead to the identification of 1) specific biomarkers mechanistically linked to metastatic disease and 2) targetable tumor dependencies that can inform novel therapeutic strategies. Prominent in this paradigm has been the use of patient-derived xenograft (PDX) mouse models for testing causal hypotheses generated from the molecular characterization of the TME in clinical samples^19^. However, a recent comprehensive analysis of PDX genomic evolution has shown that copy number alterations (CNAs) acquired during PDX passaging differed from those acquired during tumor evolution in patients, and that positive selection in humans can become dispensable during propagation in mice^20^. These findings demonstrate that genomic instability may be a previously overlooked feature of PDXs and reciprocal coevolution of tumor subclones and their microenvironments may exhibit clinically relevant differences in mice and humans^20^. Thus, stable human models recapitulating critical aspects of the TME are needed to complement mouse PDX models.

We have begun to address this unmet need by implementing a functional human Liver Acinus MicroPhysiological System (LAMPS) developed at the University of Pittsburgh Drug Discovery Institute^21,22^ that replicates some critical aspects of the liver metastatic niche. The goal is to enhance our understanding of the reciprocal relationship between ESR1 LBD mutations and the liver metastatic microenvironment, a common metastatic site for numerous types of cancer, including MBC. In this study, we have built upon our LAMPS model to quantitatively determine phenotypic differences in estrogen-dependent human cell lines (MCF7) edited to express ESR LBD mutations^14,23,24^ identified in the metastases of breast cancer patients. We examined the growth of these cells in 2D monocultures, static co-culture models and LAMPS with the latter two models containing primary human hepatocytes, together with human endothelial, Kupffer and stellate cell lines. These studies describe distinct phenotypic differences among ESR1 mutations with respect to estrogen dependence, response to changes in oxygen tension and drug resistance, indicating a key regulatory role of the TME. Controlled variation of critical parameters in evolving human liver MPS that otherwise is not easily achievable in PDX mouse models, will facilitate the identification and preclinical validation of biomarkers mechanistically linked to malignant disease progression as well as inform novel therapeutic strategies that may include agents that target specific mutant ESR1 expressing clones or that directly modify the TME.

## Results

### The liver tumor microenvironment regulates phenotypes conferred by clinically observed ESR1 mutations

**Figure 1:**
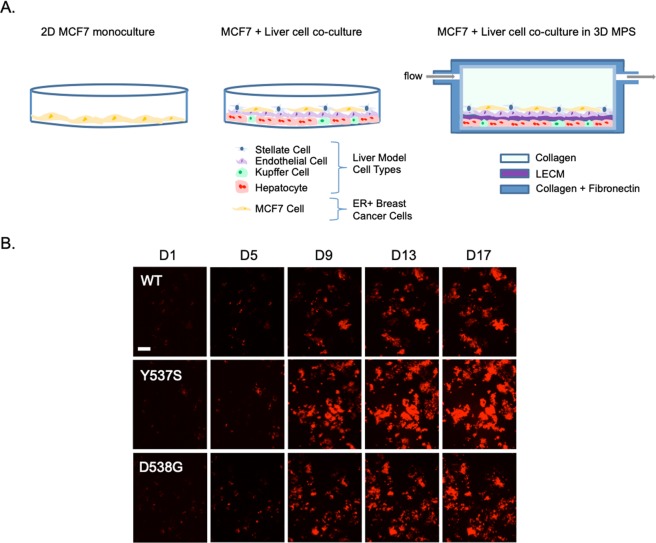
Overview of the experimental approach for studying ESR1 mutation phenotypes in the human liver microenvironment. To characterize phenotypes conferred by ESR1 mutations within the metastatic microenvironment, we examined the growth of fluorescent (mCherry-labeled) ER+ MCF7 cells that have been genome-edited to encode clinically relevant estrogen receptor LBD mutations (Y537S and D538G). We assessed the growth of these cells in 2D monoculture and static co-culture models and LAMPS with the latter two models containing primary human hepatocytes, together with human endothelial, Kupffer and stellate cell lines (**A**). Images of ESR1 wild-type or mutant-expressing cells were acquired over a 13-day (2D monoculture and static co-culture) or 17-day (LAMPS) time course. (**B**) To quantify cell growth, changes in fluorescence intensity were normalized to day 1 values for each cell line tested. Images displayed show the growth of WT and ESR1 mutant-expressing cells over a 17 day time course in the LAMPS model; scale bar 100 μm.

Our overall goal was to determine the relationships among a relevant and controllable TME of human breast cancer liver metastasis and tumor phenotypes conferred by clinically observed ESR1 mutations. To determine these relationships, we utilized fluorescent (mCherry)-labeled ER+ MCF7 cells that have been genome-edited to encode the two most common ER LBD mutations (Y537S and D538G)^14,23,24^. We studied the growth of these cells in three systems with increasing complexity to recapitulate and deconvolute aspects of the liver metastatic breast cancer niche: 2D monoculture, static co-culture model and LAMPS under flow^21^ (Fig. 1A**;** see Materials and Methods). The cell types used in both the static co-culture and LAMPS models are (Table S1): primary human hepatocytes, human endothelial, Kupffer and stellate cell lines and the number of each cell type seeded in each model. In addition, co-culture and LAMPS models include a porcine liver extracellular matrix to model the Space of Disse^21,25^, as well as a collagen I overlay to maintain hepatocyte morphology and functionality over extended culture time^22,26^. Using this experimental design, we quantitated the growth of the WT and mutant ESR1 expressing cells in the presence of estradiol (E2) or constitutively in its absence, under differing oxygen tensions, and in the presence of known ER antagonists and chemotherapeutic agents (Fig. 1A,B**)**. Initially, we examined the growth of ESR1-expressing cells in the LAMPS model and determined the spatial relationship between the cancer cells and hepatocytes. In addition to mCherry-tagged ESR1-expressing cells, we used lentiviral transduction to express GFP containing a mitochondrial targeting sequence (GFP-mito) in hepatocytes^22,27^ (see Materials and Methods), allowing us to monitor the spatial relationship between ESR1-expressing cells and hepatocytes over a 17-day time course. Using confocal analysis to generate 3D renderings for each ESR1 clone (WT, Y537S, D538G) we observe a similar spatial distribution between the hepatocyte layer and the ESR1-expressing cancer cells **(**Fig. S1A–C**)** in LAMPS models. For each ESR1-expressing clone, the peak fluorescence values are found largely within a similar z-range as the hepatocytes **(**Fig. S1D–F**)**, indicating that the cancer cells are primarily found infiltrating within the hepatocyte layer. This finding is consistent with observations made in human *in vivo* studies^28^ comparing the growth of breast adenocarcinoma liver metastases and colorectal liver metastases. Whereas the majority of breast cancer liver metastases had a replacement growth pattern in which cancer cells grow in continuity with hepatocytes, only a small percentage of colorectal metastases grew *via* this pattern. Rather, colorectal liver metastases grew in a desmoplastic pattern where the metastatic cancer cells were separated from the surrounding liver parenchyma by a layer of desmoplastic stroma.

**Figure 2:**
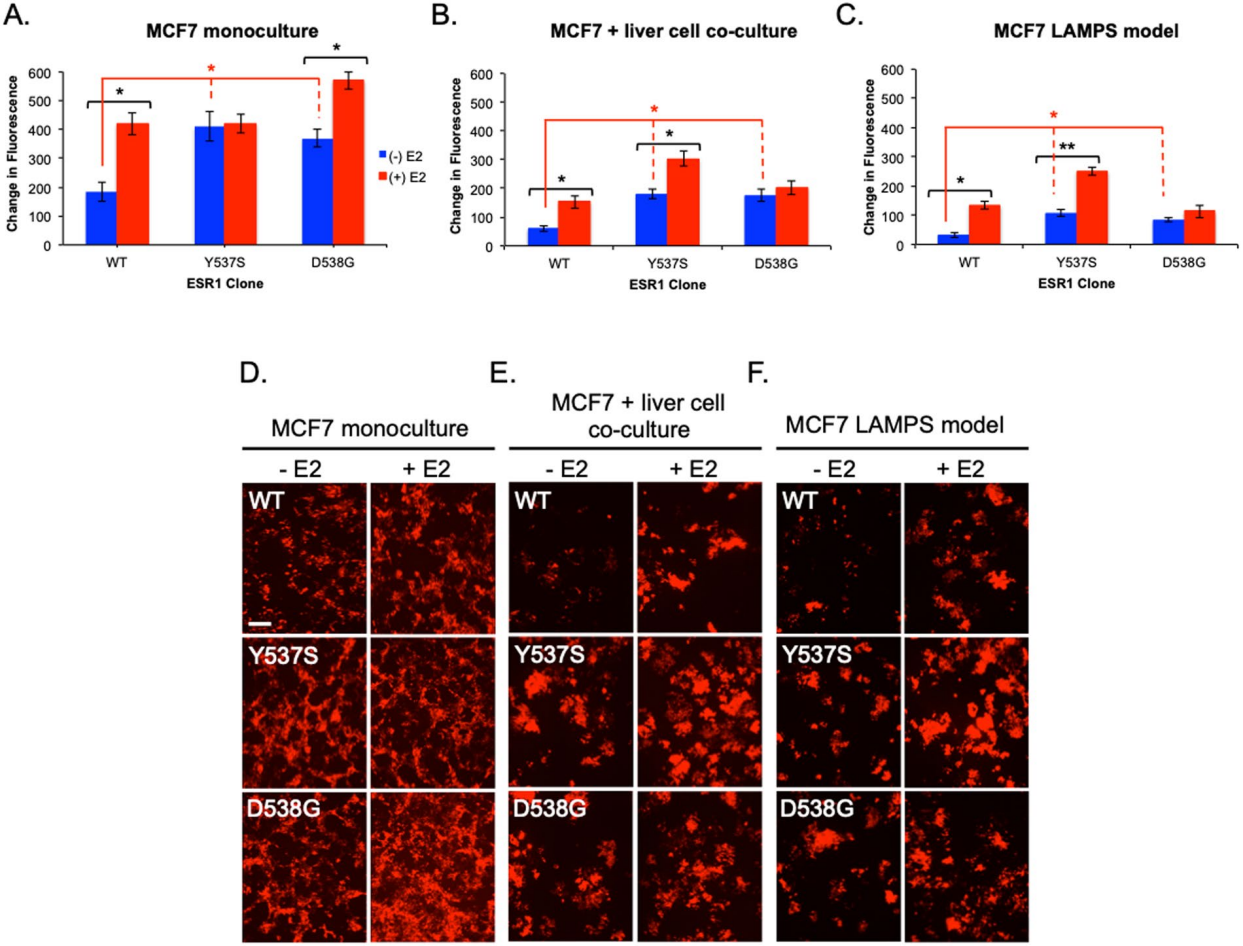
The liver TME regulates enhanced estrogen-dependent growth phenotypes conferred by clinically observed ESR1 mutations. WT, Y537S, and D538G-expressing cells were grown in the absence (blue bars) or presence of 5 nM E2 (red bars) in 2D monoculture (**A**,**D**), static co-culture (**B**,**E**), or LAMPS (**C**,**F**). Graphs are plotted as the mean change in fluorescence intensity ± SD of three fields. Experiments were repeated 3 times (n = 3) for each condition. Differences in fluorescence intensity that were reported as significant had *p ≤ *0.05 (*) or *p* ≤ 0.005 (**). For E2-dependent growth of individual clones an unpaired, 2-tailed *t*-test (black astericks) produced the following significant p-values: [monoculture; p = 0.01 (WT); p = 0.03 (D538G)], co-culture; p = 0.02 (WT); p = 0.007 (Y537S)], [LAMPS; p = 0.01 (WT); p = 0.004 (Y537S)]. A one-way ANOVA was used to compare constitutive growth of all clones [red astericks; p = 0.02 (monoculture); p = 0.01 (co-culture); p = 0.01 (LAMPS)]. ANOVA analysis across all clones treated with E2 produced significant p-values for monoculture (p = 0.04), co-culture (p = 0.02), and LAMPS (p = 0.02. One-way ANOVA analysis comparing WT clones treated with E2 compared to the constitutive growth of ESR1 mutants did not result in significant differences under any culture condition. Images were quantified by comparing the change in fluorescence intensity for WT and ESR1 mutant expressing cells after 13 days (monoculture and co-culture) or 17 days (LAMPS). Changes in fluorescence intensity were normalized to the value obtained on day 1 for each cell line. Full growth curves for each cell line and condition line are shown in Fig. S3. D-F displays representative images for each cell line −/+E2 acquired at the end of the time course. Scale bar, 100 μm. In addition to significant constitutive growth, D538G and Y537S mutations confer enhanced estrogen-dependent growth that is sensitive to the composition of the TME. While D538G clones show enhanced estrogen-dependent growth exclusively in 2D monoculture (**A**), Y537S-expressing cells display this growth advantage only in co-culture and LAMPS (**B**,**C**). Together, these results demonstrate a phenotypic switch in estrogen-dependent growth that depends upon TME composition. Similar results were also obtained using a second set of independent clones (Fig. S4).

To quantify cancer cell growth within our models, we next calibrated the imaging platform by quantifying fluorescence intensity of the respective ESR1 expressing cell lines over a 700-fold range of cell densities (200–140,000 cells; Fig. S2). Each cell line exhibited a linear increase in fluorescence and similar overall fluorescence intensities between clones at each plating density, allowing for the quantitative determination of changes in cell number over the experimental time course **(**Fig. S2**)**. To quantitate cell growth, changes in fluorescence intensity for each ESR1 clone were normalized to the starting intensity values obtained on day 1. In both co-culture and LAMPS, 300 ESR1-expressing cells were initially seeded, representing 0.1–0.3% of the total starting cell population in these models **(**Table S1**)**. For static co-culture studies, cell growth was examined over a 13-day time course where the breakdown of the collagen overlay (data not shown) appears to be a limiting factor. With the addition of flow, continuous nutrient exchange that may include the efflux of collagenases^29^ allowed for growth studies in LAMPS to be carried over an extended time course (17 days), highlighting an advantage of the microfluidic platform. Overall, the constitutive and E2-dependent growth of WT and ESR1 mutant expressing clones were approximately two-fold higher in monoculture than in the more complex models, consistent with the known suppression of tumor growth by relatively naïve cellular microenvironments^30–33^ **(**Figs 2 and S3).

The constitutive growth in each system was significantly higher for both Y537S and D538G mutants compared to WT-expressing clones **(**Fig. 2A–C; blue bars). This hallmark feature of the two mutant expressing clones was comparable to the maximally stimulated estrogen-dependent growth of the WT clone. In addition, these mutations conferred an enhanced E2-dependent growth that was sensitive to the composition of the TME Fig. 2A–C; red bars). While D538G-expressing cells showed enhanced E2-dependent growth exclusively in 2D monoculture (Fig. 2A,D), Y537S-expressing cells displayed this phenotype in both the static co-culture (Fig. 2B,E) and LAMPS (Fig. 2C,F**)** models, but not in 2D culture. In contrast, WT-expressing cells displayed E2-dependent growth in monoculture as well as co-culture and LAMPS models (Fig. 2A–C**)**, demonstrating that the basic liver TME selectively affects the enhanced E2-dependent growth phenotype of ESR1 mutants. We observe the same constitutive and E2-enhanced growth phenotypes among ESR1 clones in both static co-culture and LAMPS, which are identical in setup except that LAMPS models are maintained under continuous media flow. Thus, this demonstrates that the growth differences observed between the two ESR1 mutant clones are the result of selective effects of the liver TME, and not the presence or absence of flow. Corroborating these observations, similar constitutive and E2-enhanced growth phenotypes were observed using an independent set of WT and ESR1 mutant-expressing clones (Fig. S4**)**. Together, these results demonstrate that the ESR1 mutant-specific enhanced E2-dependent growth phenotype is context dependent both at the level of the TME composition and the specific missense mutation within the LBD.

Oxygen zonation is a critical aspect of liver function as well as a key microenvironment component in promoting metastatic disease progression^34^. Our recent studies have demonstrated in the LAMPS model system that higher oxygen tensions in zone 1 (12–15%) and lower oxygen tensions in zone 3 (3–6%) microenvironments can be established by controlling the oxygen tension in individual devices by varying the media flow rate. Rates of 15 μL/h and 5 μL/h resulted in oxygen tensions of 12–15% and 3–6%, respectively^21^. These previous studies also showed that hepatocyte albumin secretion in LAMPS devices followed a distinct pattern over time with rates reaching a maximum at six days followed by a steady decline over the next ten days. For hepatocytes in zone 1 maintained at 12–15% oxygen, albumin secretion was higher than those maintained at 3–6% oxygen, and were higher than values obtained in static co-culture^21,22,35^. Comparable patterns of albumin secretion were observed (Fig. S5) in either LAMPS or co-culture models containing WT- or ESR1 mutant (Y537S, D538G)-expressing cells indicating that with respect to albumin secretion there is no appreciable loss of hepatocyte function relative to models assembled without cancer cells at either oxygen tension^21^. In addition, hepatocyte albumin output is not differentially affected by specific ESR1 mutations within these models (Fig. S5). In addition, these results show that hepatocyte function is not overtly impacted by cancer cell growth in these models, as Y537S-expressing cells grow up to ~300 times the starting number of cells during the 13 or 17 day time course (Figures 2 and S3).

**Figure 3:**
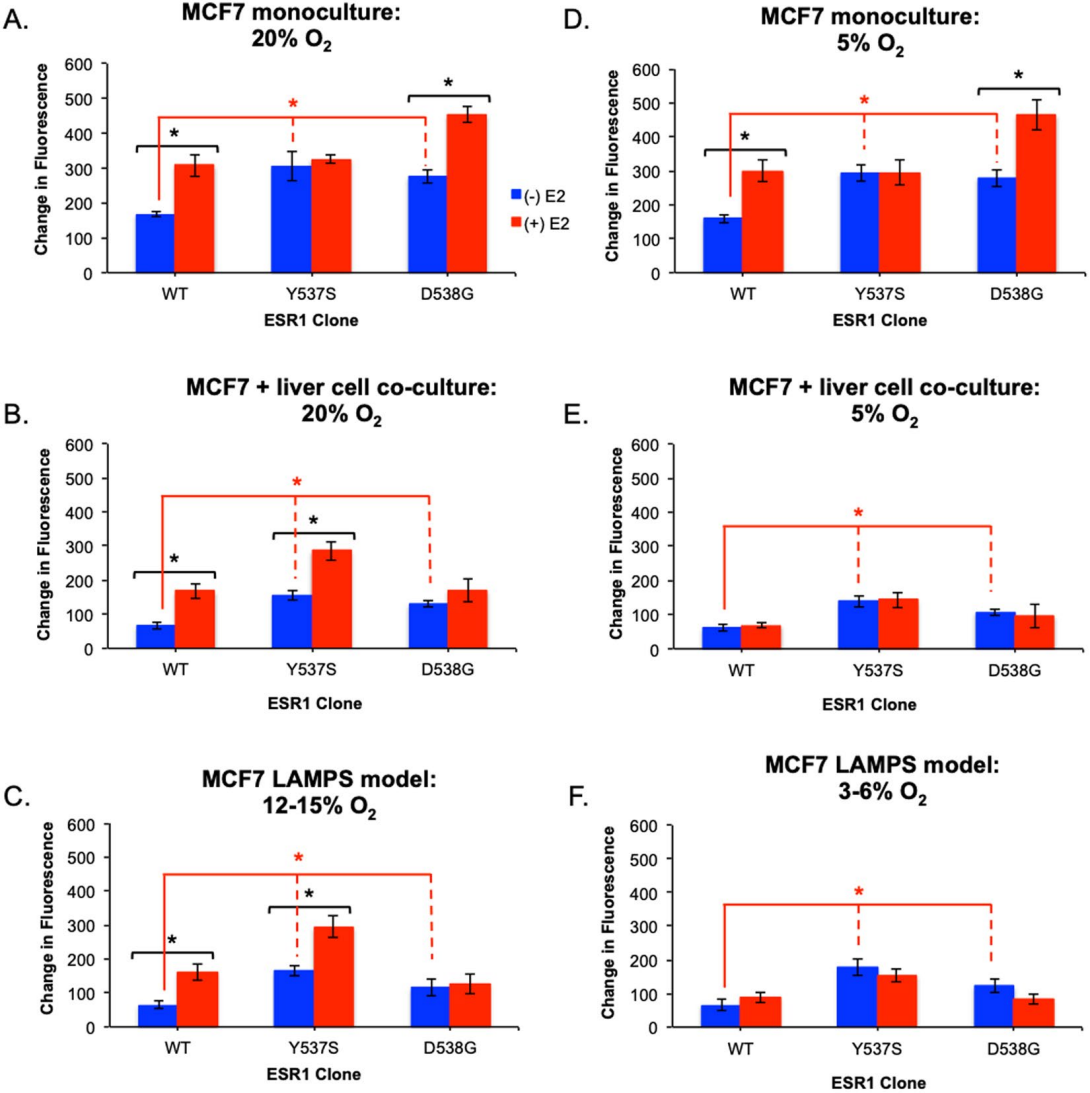
Estrogen-enhanced growth phenotypes conferred by WT and ESR1 mutations in the liver TME are regulated by oxygen tension. ESR1-expressing cells were grown in the absence (blue bars) or presence of 5 nM E2 (red bars) in monoculture, static co-culture, or LAMPS models. Monoculture and co-culture models were maintained at either 20% (**A**,**B**) or 5% (**D**,**E**) oxygen^35^, and LAMPS models were maintained at 12–15% (**C**) or 3–6% (**F**) oxygen^21^. Graphs are plotted as mean change in fluorescence intensity ± SD of three individual fields. Experiments were repeated 3 times (n = 3) for each condition. Differences in intensity measurements reported as significant had *p* ≤ 0.05 (*). For E2-dependent growth of clones maintained at Zone 1 oxygen levels an unpaired, 2-tailed *t*-test (black asterisks; **A**–**C**) produced significant p-values for: [monoculture; p = 0.02 (WT); p = 0.01 (D538G)], co-culture; p = 0.03 (WT); p = 0.02 (Y537S)], [LAMPS; p = 0.03 (WT); p = 0.01 (Y537S)]. For clones maintained at Zone 3 oxygen levels the same t-test (black asterisks; D-F) produced significant p-values for: [monoculture; p = 0.01 (WT); p = 0.008 (D538G)]. A one-way ANOVA was used to compare constitutive growth of all clones at Zone 1 oxygen tension (**A**–**C**) [red asterisks; p = 0.01 (monoculture); p = 0.02 (co-culture); p = 0.02 (LAMPS)] and Zone 3 oxygen tension (**D**–**F**) [red asterisks; p = 0.02 (monoculture); p = 0.03 (co-culture); p = 0.01 (LAMPS)]. ANOVA analysis across all clones treated with E2 produced significant p-values at Zone 1 oxygen levels (**A**–**C**) for monoculture (p = 0.01), co-culture (p = 0.02), and LAMPS (p = 0.008) as well as for cells maintained at Zone 3 oxygen tension (**D**–**F**) for monoculture (p = 0.01), co-culture (p = 0.04), and LAMPS (p = 0.04). One-way ANOVA analysis comparing WT clones treated with E2 compared to the constitutive growth of ESR1 mutants did not result in significant differences under any culture condition at Zone 1 oxygen tension (**A**–**C**). However, for cells maintained at Zone 3 oxygen tension (**D**–**F**), significant p-values were obtained in both co-culture (p = 0.04) and LAMPS (p = 0.04) models. Images were quantified as described in the legend for Fig. 2 and the Materials and Methods section. These results demonstrate the importance of oxygen tension in regulating specific growth phenotypes conferred by ESR1 mutants.

We next examined the effects of changes in oxygen tension on growth phenotypes conferred by ESR1 mutant-expressing cells in our three models. As expected, for ESR1-expressing cells maintained at zone 1 (12–15%) oxygen levels, the results mirrored the observations described in Fig. 2 and served as a control for lower oxygen tension studies. **(**Fig. 3A–C**)**. Whereas monoculture studies performed at zone 3 (3–6%) mimicked the results obtained at zone 1 (Fig. 3A,D), both co-culture and LAMPS models performed at low oxygen showed distinct differences compared to studies performed at zone 1 oxygen tension. While the constitutive growth advantage for ESR1 mutants remained unaffected, the enhanced E2-dependent growth for the Y537S mutation and the E2-dependent growth for WT expressing cells were lost. **(**Fig. 3E,F). Overall, these results demonstrate the importance of oxygen tension in regulating specific growth phenotypes conferred by ESR1 mutants. While the enhanced E2-dependent growth of the Y537S mutant and the E2-dependent growth of the WT are sensitive to changes in oxygen tension in the multicellular models, the constitutive growth maintained by the Y537S and D538G mutations and the WT are unaffected by changes in oxygen tension in the physiological range of the human liver acinus.

**Figure 4:**
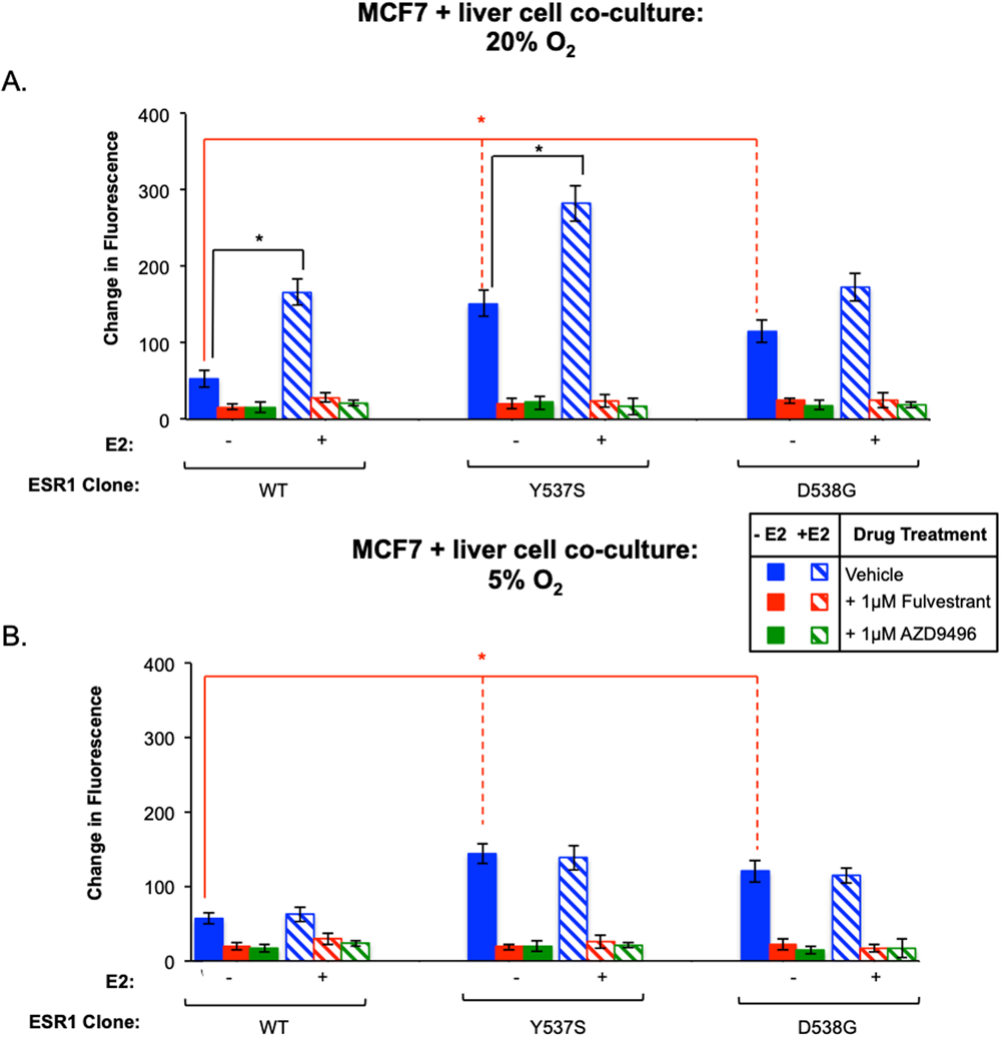
Both constitutive and estrogen-enhanced growth of ESR mutations is dependent upon ER signaling. ESR1-expressing cells were grown without (solid bars) or with 5 nM E2 (hashed bars) in co-culture, and were maintained at 20% (**A**) or 5% (**B**) oxygen for 13 days^35^. Cells were treated with vehicle (blue bars) or ER antagonists 1 μM fulvestrant (red bars) or 1 μM AZD9496. Graphs are plotted as the mean change in fluorescence intensity ± SD of three fields. Experiments were repeated 3 times (n = 3) per condition. For E2-dependent growth of individual clones maintained at Zone 1 oxygen, an unpaired, 2-tailed *t*-test (black asterisks; **A**) produced significant p-values (p < 0.05) for WT (p = 0.008) and Y537S (p = 0.01), while no significant increases were observed for clones at Zone 3 oxygen (B). A one-way ANOVA was used to compare constitutive growth of all clones at Zone 1 oxygen (**A**) [red asterisks; p = 0.01] and Zone 3 oxygen (**B**) [red asterisks; p = 0.02]. Similar analysis for clones treated with E2 produced significant p-values at Zone 1 oxygen (A; p = 0.02) as well as cells maintained at Zone 3 oxygen (B; p = 0.03). ANOVA analysis comparing WT clones treated with E2 compared to the constitutive growth of ESR1 mutants did not result in a significant difference at Zone 1 oxygen; (**A**) however, for cells maintained at Zone 3 oxygen tension (**B**), a significant difference was observed (p = 0.04). For cells maintained at 20% oxygen, in addition to significant constitutive growth observed for both mutants, Y537S mutants confer enhanced estrogen-dependent growth, consistent with the results in Fig. 3. The constitutive and estrogen-enhanced growth for both ESR1 mutants is inhibited by ER antagonist treatment, indicating dependency on ER signaling. As a control, estrogen-dependent growth of WT clones is also inhibited by ER antagonist treatment. For cells maintained at 5% oxygen (**B**), enhanced estrogen-dependent growth is lost for Y537S mutants, consistent with previous results; however, growth similar to constitutive levels is maintained and is inhibited with ER antagonist, indicating dependency on ER signaling. Although constitutive and E2-dependent growths requires ER signaling, only the E2-dependent growth is regulated by oxygen tension in co-culture and LAMPS.

As a result of the difference in oxygen sensitivity between the constitutive and E2-enhanced growth of ESR1 mutants in co-culture and LAMPS models, we next examined whether these growth phenotypes were dependent upon estrogen receptor signaling by probing for ER signaling dependency under zone 1 and zone 3 oxygen tensions using two highly selective ER antagonists having distinct chemical structures. ESR1-expressing cells grown in co-culture models in the absence (solid bars) or presence of E2 (hashed bars) were treated with vehicle control (blue bars), 1 μM fulvestrant (red bars), or 1 μM AZD9496 (green bars) and maintained in zone 1 or zone 3 conditions. These results **(**Fig. 4**)** are internally consistent with those of Fig. 3. Importantly, both constitutive and E2-dependent growth for the two mutant clones and WT clones are inhibited by either ER antagonist, indicating dependence on ER signaling. Thus, although constitutive and E2-dependent growth phenotypes require ER signaling, only the E2-dependent growth phenotypes are regulated by oxygen tension in the co-culture and LAMPS systems.

**Figure 5:**
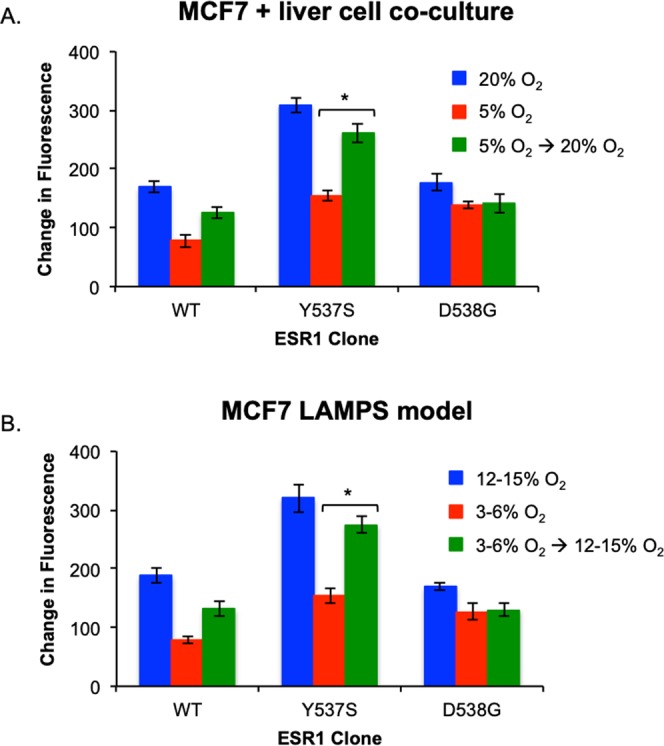
The loss of estrogen-enhanced growth for the Y537S mutation at low oxygen tension is rescued by return to higher oxygen tension. WT, Y537S, and D538G-expressing cells were grown in the presence of 5 nM E2 in both static co-culture (**A**) and LAMPS models (**B**). Cells in co-culture models were maintained for 14 days at either 20% oxygen (blue bars), 5% oxygen (red bars), or at 5% oxygen for 10 days before being moved to 20% oxygen for the remaining 4 days of the experimental time course (green bars)^35^. Similarly, for LAMPS models, cells were maintained for 14 days at either 12–15% oxygen (blue bars), 3–6% oxygen (red bars), or at 3–6% for 10 days before be changed to 12–15% oxygen for the remaining 4 days (green bars)^21^. Graphs are plotted as the mean ± SD of three fields. Experiments were repeated 3 times (n = 3) for each condition. P-values to assess significant rescue of WT or ESR1 mutant expressing cells were obtained by using an unpaired, 2-tailed t-test (*p < 0.05) where the change fluorescence intensity was normalized to the value obtained on day 1. In both co-culture and LAMPS, the enhanced E2-dependent growth observed for the Y537S mutant is lost compared to Y537S expressing cells maintained at higher oxygen levels. A similar loss of E2-dependent growth is also observed for WT expressing cells. However, for Y537S mutants cells that have been maintained in low oxygen for 10 days and then moved to higher oxygen, significant E2-enhanced growth is restored to levels comparable to that of Y537S expressing cells maintained exclusively at higher oxygen tension. While rescue of E2-dependent growth was also observed for WT cells, it was not statistically significant (*p* = 0.08). These studies demonstrate that the effect of oxygen tension on the estrogen-enhanced growth of Y537S mutant expressing cells is reversible.

We next determined if the loss of E2-enhanced growth for the Y537S mutant at zone 3(3–6%) oxygen tension could be rescued by return to culture at zone 1 (12–15%) oxygen levels in both co-culture and LAMPS models. For either model, in comparison to Y537S mutants grown exclusively at zone 3 oxygen (red bars), the E2-enhanced growth of Y537S mutant expressing cells that were returned to culture at zone 1 oxygen levels following culture at zone 3 oxygen levels (green bars), was significantly rescued to levels comparable to Y537S mutants grown exclusively at zone 1 oxygen tension (blue bars) (Fig. 5A,B). These results demonstrate that not only is the E2-enhanced growth of the Y537S mutant regulated by changes in oxygen tension, but that this regulation is also reversible. The E2-dependent growth of the WT clone showed a similar trend but did not reach statistical significance.

### The Y537S mutation displays greater resistance to fulvestrant than AZD9496 within the liver TME

**Figure 6:**
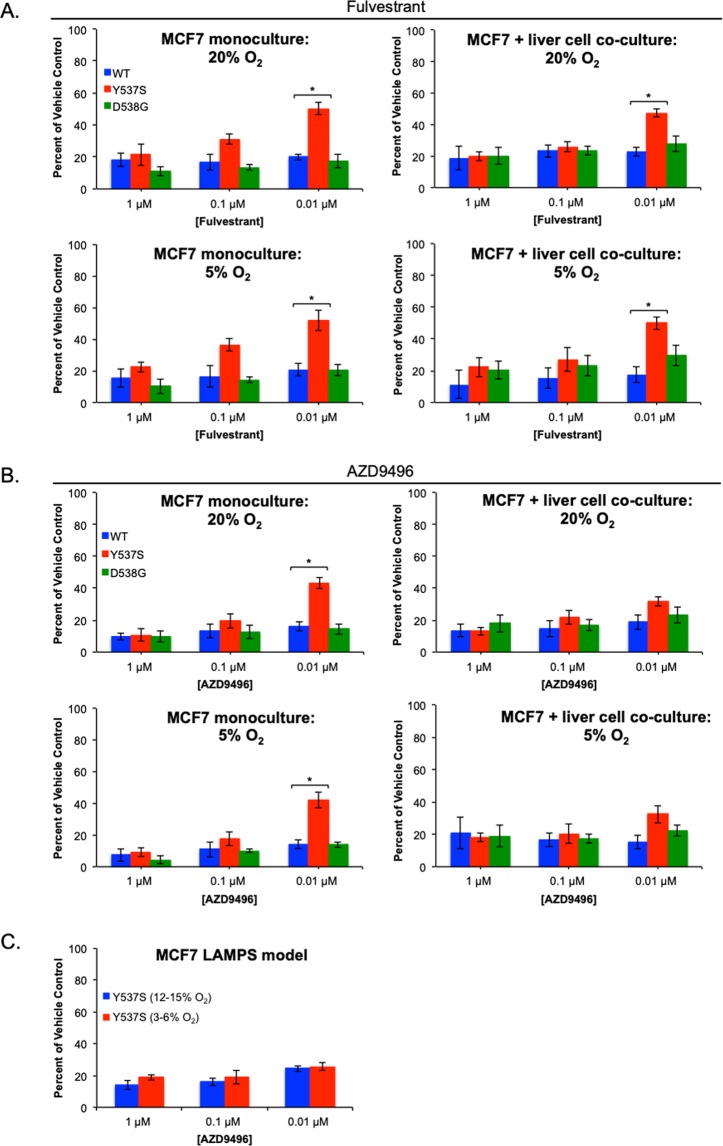
The Y537S mutation demonstrates resistance to fulvestrant in both monoculture and co-culture, but significant resistance for AZD9496 is only observed in monoculture. WT (blue bars), Y537S (red bars), and D538G (green bars) expressing cells were grown in the presence of 5 nM E2 in both monoculture and static co-culture. Cells were maintained in either 5% or 20% oxygen and were treated over a 13-day time course with the ER antagonists fulvestrant (**A**) or AZD9496 (**B**) at 1 μM, 0.1 μM, or 0.01 μM^35^. For LAMPS models (**C**), Y537S expressing were maintained at 12–15% or 3–6% oxygen tension^21^. Graphs are displayed as the percent of vehicle treated control for each ESR1 expressing cell line where the mean change in fluorescence intensity ± SD of three individual fields was quantified. Experiments were repeated 3 times (n = 3) for each condition. P-values were obtained by using an unpaired, 2-tailed t-test (*p < 0.05) to compare the growth of WT and mutant expressing cells in the presence of ER antagonist. For fulvestrant treatment, the Y537S mutation confers relative resistance at the lowest concentration of drug in both mono- and co-culture and does not vary with changes in oxygen tension of 5–20% (**A**). In contrast, the Y537S mutation confers significant resistance to AZD9496 only in monoculture, whereas in co-culture, only modest relative resistance to AZD9496 is observed compared to fulvestrant treatment at either oxygen tension (**B**). For Y537S expressing cells treated with AZD9496 in LAMPS (**C**), similar results to those obtained in co-culture were observed, suggesting that within the liver TME, AZD9496 is a more effective inhibitor of ESR1 mutant cell growth.

Previous studies have demonstrated that ESR1 LBD mutations confer resistance to ER antagonists^5,6,14,15,36–38^. Therefore, our goal was to determine whether Y537S or D538G mutants displayed resistance to treatment with ER antagonists in the context of the liver TME. We examined the growth of ESR1 mutant expressing cells at both zone 1 (12–15%) and zone 3 (3–6%) tensions upon treatment with fulvestrant and AZD9496^39^ using both monoculture and co-culture models. We focused our initial drug studies on monoculture and co-culture models, since one of the major limitations of the LAMPS model is that it has a large amount of polydimethylsiloxane (PDMS) that is known to bind hydrophobic molecules, including drugs^25^ **(**Table S2**)**. As a control, we examined the growth of these cells in the presence of doxorubicin, a known inhibitor of breast cancer cell growth in both monoculture and MPS^40–43^. As expected, neither WT nor ESR1 mutant expressing cells demonstrated resistance to doxorubicin **(**Fig. S6**)**. Upon treatment with ER antagonist, Y537S expressing cells displayed an enhanced ability to grow (~50% of vehicle control) at the lowest concentration of fulvestrant or AZD9496 in monoculture at either zone 1 or zone 3 oxygen tension, **(**Fig. 6A,B**)**, consistent with studies demonstrating ER antagonist resistance in 2D culture^14,15^. In co-culture models, Y537S mutants demonstrated statistically significant resistance to the lowest concentration of fulvestrant (~45% of control) **(**Fig. 6A**)**; however, more modest resistance to AZD9496 (~30% of control), that was not statistically significant, was observed for Y537S mutants **(**Fig. 6B**)** at either zone 1 or zone 3 oxygen tension. Similar results were also obtained for Y537S expressing cells treated with AZD9496 in LAMPS models **(**Fig. 6C; Table S2**)**^25,44–46^, suggesting that AZD9496 serves as the more effective ER antagonist to limit ESR1 mutant cell growth in the liver TME^17,41–43^.

## Discussion

The WT, Y537S, and D538G ESR1 expressing MCF7 clones exhibit both constitutive and estrogen-dependent growth phenotypes that signal through the ER **(**Fig. 4**)**. In contrast to the constitutive growth phenotypes that include the hallmark increase conferred by the two LBD mutations, one important finding of this study is the distinct differences among the estrogen-dependent growth phenotypes under cell autonomous and cell non-autonomous conditions. For the WT expressing clones, estrogen-dependent growth is evident in both the cell autonomous and cell non-autonomous models. In contrast, the D538G expressing clones exhibit an estrogen-dependent enhanced growth phenotype exclusively under cell autonomous conditions and conversely, the Y537S expressing clones display the estrogen-dependent enhanced growth only under cell-non-autonomous conditions. The results obtained in monoculture mirror those obtained in our previous cell autonomous studies with another breast cancer parental cell line, T47D, edited to express the same two ESR1 LBD mutations^15^. These results suggest that the estrogen-enhanced growth of D538G expressing MCF7 cells is suppressed through heterotypic signaling within a cell non-autonomous niche that conversely, can potentiate estrogen-enhanced growth of Y537S expressing cells. Thus, implementation of these cell non-autonomous models has enabled the detection of differential intercellular regulation of distinct growth phenotypes that could support clonal selection and determine the mutational landscape within particular metastatic sites.

The faithful recapitulation of liver acinus metabolic zonation oxygen levels within our MPS^21^ has also enabled us to study the dependency of WT and mutant ESR1-conferring growth phenotypes on the physiologically relevant range of molecular oxygen levels (Fig. 3). Under the cell autonomous condition there was no significant effect of molecular oxygen in the range of 5–20% on the constitutive and estrogen-dependent growth phenotypes of the ESR1 clones. However, under cell non-autonomous conditions, the responses of constitutive and estrogen-dependent growth to changes in molecular oxygen levels were distinct. While the constitutive growth phenotypes for the three ESR1 clones remained invariant upon changes in oxygen levels, the estrogen-dependent growth phenotype of the WT clone and the enhanced estrogen-dependent growth phenotype of the Y537S expressing clone were lost at the zone 3 (3–6%) oxygen levels. The constitutive growth of the mutant ESR1-expressing clones is comparable to maximally estrogen-stimulated growth of the WT expressing-clones. Therefore, the constitutive growth conferred by mutant ESR1 may impart a growth advantage compared to WT expressing clones not only at low estrogen levels (i.e., during estrogen deprivation therapy) but also at sites where oxygen may be limiting (i.e., within primary tumors providing positive selection of minor clones with metastatic potential and within nascent metastatic sites, potentially initiated in zone 3). Several reports suggest that hypoxia and estrogen signaling pathways converge on histone demethylases^47–49^. In particular, the alpha-glutarate-dependent dioxygenase KDM4B (aka, JMJD2B) has been shown to be important in G2/M progression and ER+ breast tumor growth in several models that include MCF7^50–53^. Since the lysine demethylase activity of KDM4B is dependent on molecular oxygen, it seems plausible that correspondingly reduced KDM4B enzymatic activity at lower levels of oxygen could contribute to the loss of estrogen-dependent growth phenotypes observed in our study.

Differences between the *in vitro* cell culture environment and the *in vivo* environment have prompted the development of patient-derived xenograft (PDX) models to complement simpler cell-line-based models in order to recapitulate critical aspects of human tumor biology. However, recent studies suggest that PDX passaging leads to mouse–specific tumor evolution^20^. For example, several copy number alterations recurrently observed in human primary tumors, indicative of positive selection in patients, gradually disappeared in PDXs^20^. Therefore, it is important to complement these existing models with ones that mirror clinically significant aspects of human tumor biology. The study presented here demonstrates the use of a human liver MPS to determine the regulation by the cell non-autonomous tumor microenvironment of ER+ breast cancer metastatic phenotypes conferred by specific ESR1 mutations. The cancer cells in the LAMPS model display a growth pattern that is largely consistent with a previous human *in vivo* study examining the growth of characteristics of metastatic breast cancer cells within the liver^28^. Similar to these results, MCF7 cells in LAMPS grow within and in close proximity to hepatocytes in the early growth of the tumor, indicative of a replacement-like growth pattern^28^. Consistent with the known suppression of tumor growth by the TME, both the constitutive and E2-dependent growth of WT and ESR mutant expressing cells were two-fold higher in monoculture compared to co-culture and LAMPS models^30,31^. Our findings demonstrate constitutive growth activity for both the Y537S and D538G mutants in monoculture as well as in both co-culture and LAMPS models, consistent with observations made for these mutants in both *in vitro* culture and *in vivo* MCF7 xenograft models.^5,6,14,15,36,37,54,55^. Similar to *in vivo* MCF7 xenograft studies, ESR1 WT clones in both co-culture and LAMPS models display estrogen dependent growth at an oxygen tension of 12–15%^56^. However, a unique finding of this study is the estrogen-enhanced growth of the Y537S mutant in both co-culture and LAMPS models at zone 1 oxygen levels (12–15%). Moreover, the estrogen enhanced growth for both WT and Y537S expressing clones is lost at lower oxygen tension of 3–6% found in zone 3 of the liver acinus, highlighting the selective role of the liver TME in regulating growth phenotypes conferred by both WT and ESR1 mutant clones in our study. Finally, with respect to the effects of ER antagonist treatment on the growth of ESR1 mutant expressing cells, our results are consistent with MCF7 xenograft models that show AZD9496 to be a more effective inhibitor of tumor growth *in vivo* compared to fulvestrant while also demonstrating increased resistance of the Y537S mutation to is ER antagonist treatment compared to the D538G mutation^37,39,55^.

As discussed above, these studies describe novel phenotypic differences compared to monoculture among ESR1 mutations with respect to estrogen-dependence, changes in oxygen tension, and drug resistance using all-human co-culture and LAMPS models. As xenograft models contain limitations (lack an intact immune system, genomic instability, and the presence of mouse stromal components), the use of all-human model systems is an important complementary approach for studying the role of the TME in cancer metastasis. Although in these studies we observe similar results between co-culture and LAMPS models, the development of MPS models to study the metastatic process is an iterative process. Recent advances in the liver MPS (vLAMPS) that include vascularization, creation of continuous oxygen zonation, the use of glass to replace PDMS to permit drug studies, the coupling of two or more organs-on-a-chip^57–59^, and the incorporation of immune cells^25^ support the prospect of applying these MPS models to probe disease progression. Furthermore, the advances provide an improved platform for modeling metastatic events (extravasation; dormancy; immune cell infiltration) within the context of the tumor microenvironment^25,41,43,60–62^. Further advances in the vLAMPS will also include the incorporation of adult human iPSC-derived cells from individual patients to replace human primary cells and cell lines. Further development is required, but success with generating adult iPSC-derived liver cells will create personalized liver MPS that will permit the incorporation of patient tumor cells, as well as the full use of innate and adaptive immune cells in the vascular system. Continued evolution of the biomimetic characteristics of vLAMPS will also include the incorporation of bile ducts and coupling with other critical organ MPS for specific applications.

We envisage the use of personalized liver MPS models for identifying biomarkers mechanistically linked to the patient’s pathophysiology that, in turn, will inform optimal therapeutic strategies for individual patients. The use of hyperplexed (use of >7) fluorescence-based biomarkers in the same tissue^63^ has accelerated the development of computational pathology tools to understand the heterogeneity within TMEs. This information is used as a guide to optimize the physiological relevance of TME’s engineered into MPS models and to develop systems biology models of tumor progression and predictive models of cancer recurrence^63–65^.

## Materials and Methods

### Reagents

Fulvestrant, doxorubicin, phorbol myristate acetate, fibronectin, and β-estradiol (E2) were obtained from Sigma Aldrich (St. Louis, MO). AZD9496 was obtained from MedChemExpress (Monmouth Junction, NJ). Recombinant human vascular endothelial growth factor (VEGF) was obtained from ThermoFisher (Waltham, MA). Rat-tail collagen type 1 was purchased from Becton Dickinson (Franklin Lakes, NJ).

### Cell sources and culture

A single lot of selected cryopreserved primary human hepatocytes (lot# Hu8241) with >90% viability and re-plating efficiency post-thaw were purchased from ThermoFisher. Human dermal microvascular endothelial cells (HMVEC-D) were purchased from Lonza (CC-2505, Alpharetta, GA). The human monoblast cell line, THP-1, used to generate Kupffer cells, was purchased from ATCC (Rockville, MD). LX-2 human stellate cells were acquired from EMD Millipore (Billerica, MA). The LX-2 cell is an immortalized human hepatic stellate cell that constitutively expresses key receptors regulating hepatic fibrosis, and proliferates in response to PDGF, a prominent mitogen contributing to liver fibrosis^66,67^. Genome-edited MCF7 cell lines were generated and characterized as previously described^14,23,24^, and were provided by Ben Ho Park (The Johns Hopkins University School of Medicine, Baltimore, MD) and the mCherry-tagged counterparts by Steffi Oesterreich and Adrian Lee (University of Pittsburgh, Pittsburgh, PA).

Primary human hepatocytes were cultured in hepatocyte maintenance media (HMM) consisting of 96% Williams E medium (ThermoFisher), 4% cocktail B (ThermoFisher, CM4000), and 100 ηM Dexamethasone (ThermoFisher). LAMPS perfusion media contained HMM, 1% charcoal stripped serum (CSS; ThermoFisher), 10 ηg/mL human recombinant VEGF (ThermoFisher), and 50 μg/mL porcine liver extracellular matrix^68^ (LECM; provided by Dr. Stephen Badylak’s laboratory, University of Pittsburgh). All perfusion media was equilibrated to atmospheric oxygen containing 18% dissolved oxygen^69–71^. HMVEC-D cells were cultured in endothelial cell basal medium-2 (EBM-2) supplemented with the endothelial growth medium-2 (EGM-2) supplement pack (Lonza). THP-1 cells were cultured in suspension in RPMI-1640 medium (ThermoFisher) supplemented with 10% fetal bovine serum (FBS; ThermoFisher), 100 μg/mL penicillin streptomycin (ThermoFisher), and 2 mM L-glutamine (ThermoFisher). THP-1 cells were differentiated into mature macrophages by treatment with 200 ηg/mL phorbol myristate acetate (Sigma Aldrich) for 48 h. Differentiated THP-1 monocytes release human tumor necrosis factor alpha (TNF-α) and interlukin-6 (IL-6) in response to LPS treatment, a condition reported to induce the immune mediated liver toxic response in *in vitro* models^22,72,73^. LX-2 cells were cultured in Dulbecco’s Modified Eagle Medium (DMEM; ThermoFisher) supplemented with 2% FBS and 100 μg/mL penicillin streptomycin. MCF7 cells were cultured in Minimum Essential Medium (MEM; ThermoFisher) supplemented with 10% FBS (ThermoFisher), 100 μg/mL penicillin streptomycin (ThermoFisher), and 0.1 mg/ml recombinant human insulin (ThermoFisher). For estrogen deprivation studies, MCF7 cells were transferred from complete media to estrogen-free media composed of phenol-free Improved Minimal Essential Medium (IMEM; ThermoFisher) supplemented with 5% CSS and 100 μg/mL penicillin-streptomycin.

### LAMPS assembly and maintenance

Human Liver Acinus MicroPhysiology System (LAMPS) studies were carried out as previously described^21^ with modification to include the addition of MCF7 cells. A single chamber commercial microfluidic device (HAR-V single channel device, SCC-001, Nortis, Inc. Seattle, WA) was used for LAMPS studies. The devices were stored in phosphate-buffered saline (PBS) until use. The interior of the devices was dried under vacuum prior to protein coating with 100 μg/mL bovine fibronectin and 150 μg/mL rat-tail collagen, type 1, alpha 2 in PBS as previously described^22^. For all steps involving injection of media and/or cell suspensions into LAMPS devices, 100–150 μl per device was used to ensure complete filling of fluidic pathways, chamber and bubble traps. On the first day of LAMPS assembly, cryopreserved hepatocytes were thawed following the manufacturer’s recommendations. Hepatocytes were pelleted at 100 x g for 3 min, re-suspended at 2.75 × 10^6^ hepatocytes/mL in plating media containing Williams E medium supplemented with 5% fetal bovine serum, 100 mg/mL penicillin-streptomycin and 2 mM L-glutamine (hepatocyte plating media), then injected into the interstitial compartment of the device for overnight incubation at 37 °C to allow adherence and spreading. On the second day of LAMPS assembly, plating media was removed from the device and a solution of 400 μg/ml of porcine LECM (a kind gift from Dr. Stephen Badylak’s laboratory at the McGowan Institute for Regenerative Medicine, University of Pittsburgh) was added and incubated for 3 h at 37 °C to create a thin matrix layer on top of the hepatocytes. The addition of LECM on the top of the hepatocyte layer serves to mimic the Space of Disse—a protein rich interface that creates a permeable layer in the sinusoidal lumen between the endothelial and hepatic compartments^21^. A suspension of 3.0 × 10^6^ HMVEC-D cells/mL, 0.8 × 10^6^ differentiated THP-1 cells/mL, and 3 × 10^3^ MCF7 cells/mL in HMM was then injected on top of the LECM, and incubated for 2 h at 37 °C before the final injection of 0.2 × 10^6^ LX-2 cells suspended in 1 mL of a 2.5 mg/mL solution of pH 7.2 rat-tail collagen/10 mM HEPES/HBSS. The collagen overlay functions to maintain hepatocyte morphology and functionality over extended culture time^22,26^. The percentages of THP-1, HMVEC-D, and LX-2 cells are consistent with the scaling used in our previously published models (Table S1**)**^21,22^. The devices were inverted for 1 h at 37 °C during collagen polymerization to ensure an initial spatial separation of hepatocytes and LX-2 stellate cells. The devices were re-inverted and incubated overnight to allow stabilization of the model before initiating 5 (3–6% O_2_) and 15 (12–15% O_2_) μL/hour perfusion with HMM supplemented with 10 ηg/mL VEGF, 100 ηM dexamethasone and 50 mg/mL of porcine LECM at 37 °C in 5% CO_2_ humidified atmosphere. The perfusion rates required to achieve target oxygen concentrations were determined as previously described^21^. The flow rates of 5 μl/hr and 15 μl/hr result in respective volumes of 120 μl and 360 μl of media flowing through the device per day. The total volume required to fill a LAMPS device is ~100 μl; therefore, at a flow rate of 5 μl/hr, the total fluid volume is replaced once per day and at a flow rate of 15 μl/hr, the total fluid volume is replaced three times per day. Perfusion media was then prepared according to the experimental layout (−/+E2; −/+fulvestrant, AZD9496, or doxorubicin) and flow was then initiated (day 0) to start the experimental time course. β-estradiol was solubilized in ethanol and added to the appropriate cells at a final concentration of 5 ηM; Fulvestrant, AZD9496, and doxorubicin were solubilized in DMSO and added to cells at the appropriate concentration (1 μM, 0.1 μM, or 0.01 μM). The final concentration of ethanol or DMSO for vehicle control treatment was 0.08% (v/v).

### Monoculture and co-culture model assembly and maintenance

For monoculture studies, 300 MCF7 cells per well were plated into a collagen I-coated clear-bottom 96 well plate (354649; ThermoFisher) in HMM containing 1% CSS, 10 ηg/mL human recombinant VEGF (ThermoFisher), and 50 μg/mL porcine LECM. For co-culture studies, 50,000 hepatocytes per well were plated in a clear-bottom 96 well plate with hepatocyte plating media and incubated overnight at 37 °C to generate a confluent monolayer of cells (Table S1). The next day, plating media was removed from the wells and 80 μl of 400 μg/ml of porcine LECM solution prepared in HMM containing 1% CSS was added to each well and incubated for 3 h at 37 °C to create a thin matrix layer on top of the hepatocytes. A suspension of 0.54 × 10^6^ HMVEC-D cells/mL, 0.28 × 10^6^ differentiated THP-1 cells/mL, and 5 × 10^3^ MCF7 cells/mL in HMM + 1% CSS was prepared and 100 μl per well was gently seeded on top of the LECM, and incubated for 2 h at 37 °C to allow cells to attach. The media from the wells was gently removed by inverting the plate before overlaying 40 μl of 0.1 × 10^6^ LX-2 cells suspended in 1 mL of a 2.5 mg/mL solution of pH 7.2 rat-tail collagen/10 mM HEPES/HBSS. The plates were incubated upright for 10 minutes to begin to allow the collagen to polymerize before being inverted for an additional 1 h at 37 °C to allow for full collagen polymerization to ensure an initial spatial separation of hepatocytes and LX-2 stellate cells. Plates were then returned to the upright position and 60 μl of HMM + 1% CSS was added to each well, and cells were incubated overnight at 37 °C. The next day, the media from overnight culture was gently removed and both monoculture and co-culture models were maintained in 100 μl LAMPS perfusion media maintained (day 0) at 5% or 20% oxygen using a previously described method for exposing cells to normoxic and hypoxic conditions using vacuum bags to establish and maintain low oxygen levels *in vitro*^35^. Perfusion media containing E2 and/or drug treatment was prepared as described above and was gently removed and replaced every 48 h during the experimental time course.

### Image acquisition and analysis

Imaging experiments to assess MCF7 cell growth were performed with a Lumascope LS720™ inverted microscope (Etaluma, Inc., Carlsbad, CA) using a Motic EF-N Plan 4x objective (NA = 0.1) and the complementary Lumaview 720/600-Series™ software. Images for each mCherry-tagged ESR1 expressing cell line were acquired using the same exposure settings (exposure time, gain, illumination percent) and filter set (excitation 580–598 ηm; emission 612–680 ηm) using a monochrome CMOS Sensor 5-megapixel camera. The captured images for each cell line were collected at days 1, 5, 9, and 13 for monoculture and co-culture experiments, and at days 1, 5, 9, 13, and 17 for LAMPS studies. Image analysis was performed using Fiji (ImageJ; rsb.info.nih.gov/ij)^74,75^ with an intensity threshold of 20 to exclude background fluorescence to evaluate changes in fluorescence intensity (arbitrary units; AU). Changes in fluorescence intensity over time were normalized to intensity values obtained on day 1.

### Statistics

The data are presented as the mean change in fluorescence intensity ± standard deviation (SD) for 3 individual plate wells or for 3 individual fields captured per LAMPS device. Each individual 96-well plate and LAMPS experiment was repeated three times. P-values were obtained comparing the change in fluorescence intensity for WT and ESR1 mutant expressing cells after 13 days (2D monoculture and co-culture models) or 17 days of culture (LAMPS models). Differences in fluorescence intensity measurements reported as significant had *p ≤ *0.05 (*) or *p ≤ *0.005 (**) for all statistical tests. The application of an unpaired, 2-tailed *t*-test with the assumption of equal variance was used for comparisons made within the same clone to compare the growth of wild-type, Y537S, or D538G-expressing cell treated with (+E2) and without (−E2) estrogen. For statistical comparisons made across all clones, a one-way ANOVA was used to assess significance in constitutive (−E2) growth in ESR1 clones, to compare the estrogen-enhanced (+E2) growth of the wild-type clone to the constitutive (−E2) growth of ESR1 mutant clones, and to compare the estrogen-enhanced growth of ESR1 clones.

## Supplementary information

Supplementary Information

## Data Availability

The datasets generated and analyzed during the current study are available from the corresponding author on reasonable request.
