# Supplementary material for: Modeling the Effect of the Metastatic Microenvironment on Phenotypes Conferred by Estrogen Receptor Mutations Using a Human Liver Microphysiological System
Source: Sci Rep. 2019 Jun 6;9:8341. doi: 10.1038/s41598-019-44756-5 (PMC6554298; doi:10.1038/s41598-019-44756-5)
Supplement: Supplementary file 1 — Supplementary Information [file 41598_2019_44756_MOESM1_ESM.pdf]

Supplementary Material

Modeling the Effect of the Metastatic Microenvironment on Phenotypes Conferred by Estrogen Receptor Mutations Using a Human Liver Microphysiological System.

Mark T. Miedel[1, 2], Dillon C. Gavlock[1, 2], Shanhang Jia[1, 3], Albert Gough [1, 2], D. Lansing Taylor[1, 2, 4 *], Andrew M. Stern[1, 2 *]

[1] Drug Discovery Institute, University of Pittsburgh, Pittsburgh, PA, USA

[2] Department of Computational and Systems Biology, University of Pittsburgh, Pittsburgh, PA, USA

[3] School of Medicine, Tsinghua University, Beijing, China

[4] University of Pittsburgh Cancer Institute, Pittsburgh, PA, USA

* sternam@pitt.edu; dltaylor@pitt.edu

Supplemental Materials and Methods

Lentiviral transduction of hepatocytes and confocal imaging to examine the spatial relationship between MCF7 cells and hepatocytes. The hepatocyte biosensor construct pCT-mito-GFP was obtained from Systems Biosciences (Mountain View, CA) and contains a mitochondrial localization tag derived from cytochrome C that is C-terminally fused to GFP. Lentiviral particles were produced and transductions performed as previously described [1,2]. To examine the spatial relationship between hepatocytes and mCherry-tagged ESR1-expressing mutant cells, LAMPS models were set up as described in the Materials and Methods with the additional step of hepatocyte transduction. Briefly, after thaw, hepatocytes were incubated for 2h in lentivral supernatant generated from pCT-mito-GFP diluted to an MOI of 5 and containing 8μg/mL polybrene (EMD Millipore). Following this incubation, hepatocytes were gently pelleted, resuspeded, and seeded into LAMPS models as described in the Materials and Methods. Hepatocytes reached their maximum transduction efficiency (~50%) after 72h and GFP expression remained stable throughout the experimental time course. Once models were assembled and flow (15 μl/h) established, images were collected with a Nikon 20x (0.45 NA) objective using the IN Cell Analyzer 6000 (GE Healthcare) in confocal mode using the 488 nm (GFP) and 561 nm (mCherry) lasers and associated emission filter sets with the aperture set to 1 airy unit. Z-stacks were collected after days 1, 9, and 17 of the experimental time course. During imaging, 35 z-slices were acquired for each field with 3μm spacing between slices (105 μm distance). For each ESR1 clone, 3 individual LAMPS models were imaged, acquiring 8 fields per device. Image

stacks were then imported into ImageJ (FIJI) to generate 3D renderings and orthogonal view images to describe the spatial relationship between hepatocytes and MCF7 cells. Fluorescence intensity in individual z-planes was analyzed in ImageJ and intensity values were normalized to the highest peak intensity in each plane of the stack and were plotted for each individual z-slice. For statistical analysis comparing fluorescence within the z-stack between hepatocytes and MCF7 cells, a Kruskal-Wallis rank sum test was used to assess significant differences in intensity throughout the z-stack. For each ESR1 clone 3 individual LAMPS models were evaluated (8 fields/device). For WT, Y537S, and D538G LAMPS models P-values of 0.23, 0.31, and 0.27 were obtained, respectively.

Fluorescence intensity calibration

For each mCherry-tagged ESR1 expressing cell lines varying cell concentrations were seeded (200, 500, 1,000, 5,000, 10,000, 20,000, 40,000, 80,000, 100,000, 120,000, 140,000 cells/well) into triplicate wells of a clear bottom 96-well plate. Cells were incubated for 2 hours at 37°C allowing for cellular adhesion, then labeled with 1µg/mL Hoechst 33342 (Invitrogen) for 15 minutes. Subsequently, images were collected as described above with a Lumascope LS720™ inverted microscope. Images for each ESR1 expressing cell line were acquired using filter sets to detect mCherry (excitation 580-598, ηm emission 612-680 ηm) and Hoechst (excitation 370-410 ηm, emission 429-462 ηm). The captured images were analyzed using ImageJ to identify cell counts and mCherry average fluorescence intensity per well (n = 3 wells) as previously described[3]. The slopes obtained for each of the cell lines varied by < 10% and were given by the

equation for each line: (WT: y = 6.8x + 39284; Y537S: y = 6.5x + 22421; D538G: y = 7.0x + 35231). A one-way ANOVA test performed comparing the fluorescence intensities for ESR1 clones at the indicate cell densities. The results of this analysis produce P-values > 0.2 for each cell density, indicating that there is no statistical difference in the average fluorescence intensity between the WT, Y537S, and D538G clones at any of the plating densities examined.

Sample collection and albumin measurement

Efflux media collection and albumin measurements were performed as previously described [2,4] using an enzyme-linked immunosorbent assay (ELISA) (Bethyl Laboratories, Montgomery, TX) according to the manufacturer's specifications in a 96-well plate format.

Drug binding/recovery in PDMS-containing LAMPS device

To assess the drug binding capability of the polydimethylsiloxane (PDMS)-containing LAMPS device for compounds used in these studies (β-estradiol, fulvestrant, AZD9496, and doxorubicin), we used perfusion flow tests and mass spectrometry analysis of efflux to determine the overall effective concentration of each compound as previously described [2,5]. These studies provided the basis for the use of the monoculture and co-culture models for our initial drug testing studies described in the Results section.

Supplemental References

1 Senutovitch, N. *et al.* Fluorescent protein biosensors applied to microphysiological systems. *Exp Biol Med (Maywood)* **240**, 795-808, doi:10.1177/1535370215584934 (2015).

2 Vernetti, L. A. *et al.* A human liver microphysiology platform for investigating physiology, drug safety, and disease models. *Exp Biol Med (Maywood)* **241**, 101-114, doi:10.1177/1535370215592121 (2016).

3 Schindelin, J. *et al.* Fiji: an open-source platform for biological-image analysis. *Nat Methods* **9**, 676-682, doi:10.1038/nmeth.2019 (2012).

4 Lee-Montiel, F. T. *et al.* Control of oxygen tension recapitulates zone-specific functions in human liver microphysiology systems. *Exp Biol Med (Maywood)* **242**, 1617-1632, doi:10.1177/1535370217703978 (2017).

5 Li, X., George, S. M., Vernetti, L., Gough, A. H. & Taylor, D. L. A glass-based, continuously zonated and vascularized human liver acinus microphysiological system (vLAMPS) designed for experimental modeling of diseases and ADME/TOX. *Lab Chip*, doi:10.1039/c8lc00418h (2018).

6 Stessels, F. *et al.* Breast adenocarcinoma liver metastases, in contrast to colorectal cancer liver metastases, display a non-angiogenic growth pattern that preserves the stroma and lacks hypoxia. *Br J Cancer* **90**, 1429-1436, doi:10.1038/sj.bjc.6601727 (2004).

7 Bakmiwewa, S. M., Heng, B., Guillemin, G. J., Ball, H. J. & Hunt, N. H. An effective, low-cost method for achieving and maintaining hypoxia during cell culture studies. *Biotechniques* **59**, 223-224, 226, 228-229, doi:10.2144/000114341 (2015).

Supplemental Figure Legends

Figure S1. ESR1 mutants grow largely within the hepatocyte layer in LAMPS models. To determine the spatial relationship between the cancer cells and hepatocytes were transduced to express GFP containing a mitochondrial targeting sequence (GFP-mito), allowing for the examination of the spatial relationship between GFP-expressing hepatocytes and mCherry-expressing MCF7 cells over a 17-day time course. (A-C) For each ESR1 clone (WT, Y537S, D538G), MCF7 cells (red) were found

infiltrating within the hepatocyte (green) layer, consistent with previous reports describing the growth characteristics of metastatic breast cancer cells within the liver[6]. For each ESR1-expressing clone, the peak fluorescence values are found within a similar z-range as the hepatocytes (D-F), indicating that MCF7 cells are growing within the hepatocyte layer, with some of the Y537S and the D538G also growing slightly above the hepatocyte layer as indicated in the images (B, C – Day 16) and the shoulders around Z=65-85 μm on the graphs (E, F). A Kruskal-Wallis rank sum test was used to assess significant differences in intensity throughout the z-stack between hepatocytes and each ESR1 clone, and no significant differences were observed. For each ESR1 clone 3 individual LAMPS models were evaluated (8 fields/device). For WT, Y537S, and D538G LAMPS models P-values of 0.23, 0.31, and 0.27 were obtained, respectively. Z-stacks were acquired on Days 1, 9, and 17. Scale bar; 200 μm.

Figure S2. Calibration demonstrates a linear increase in fluorescence intensity at varying plating densities of ESR1 expressing cell lines. For each ESR1 expressing cell line, cells were seeded into a 96-well plate and were allowed to attach for 2 h at 37°C. Cells were then labeled with Hoechst for 15 min. to label nuclei and were imaged using the Lumascope LS720™ platform as described in the Materials and Methods section. For each individual cell line, fluorescence intensity increased in a linear manner (R^2 value > 0.95) with an increasing number of cells and did not substantially vary between clones at each plating density. The slopes obtained for each of the cell lines varied by < 10% and were given by the equation for each line: (WT: $y = 6.8x + 39284$; Y537S: $y = 6.5x + 22421$; D538G: $y = 7.0x + 35231$). A one-way ANOVA

test was performed comparing the fluorescence intensities for ESR1 clones at the indicate cell densities. The results of this analysis produce P-values > 0.2 for each cell density, indicating that there is no statistical difference in the average fluorescence intensity between the WT, Y537S, and D538G clones at any of the plating densities examined. The data are displayed as the average ± SD fluorescence intensity per well (n = 3 wells) for each cell line and plating density. The table below shows the average fluorescence intensity (AU) for each of the cell densities examined in the calibration curve for each ESR1-expressing cell line.

Figure S3. The liver TME regulates enhanced estrogen-dependent growth phenotypes conferred by clinically observed ESR1 mutations. Growth curves over a 13-day or 17-day time course are shown for WT (blue), Y537S (red), and D538G (green) expressing cells grown in the absence (solid lines) or presence of 5 nM E2 (broken lines) in either 2D monoculture (A), static co-culture (B), or LAMPS (C) models. Changes in fluorescence intensity were normalized to the value obtained on day 1 for each cell line and plotted over time. As described in Figure 2, D538G clones show enhanced estrogen-dependent growth exclusively in 2D monoculture (A), Y537S-expressing cells display this growth advantage only in co-culture and LAMPS (B, C). Together, these results demonstrate a phenotypic switch in estrogen-dependent growth that depends upon TME composition.

Figure S4. Enhanced estrogen-dependent growth phenotypes are conferred in a similar manner in a second set of WT and ESR1 mutant-expressing cell lines. A

second set of independent clones of WT, Y537S, and D538G-expressing cells were grown in the absence (blue bars) or presence of 5 nM E2 (red bars) in either 2D monoculture (A), static co-culture (B), or LAMPS (C) and were quantified as described in Figure 2. For E2-dependent growth of individual clones an unpaired, 2-tailed *t*-test (black asterisks) produced the following significant p-values: [monoculture; p=0.02 (WT); p=0.02 (D538G)], co-culture; p=0.04 (WT); p=0.01 (Y537S)], [LAMPS; p=0.01 (WT); p=0.008 (Y537S)]. A one-way ANOVA was used to compare constitutive growth of all clones [red asterisks; p=0.01 (monoculture); p=0.01 (co-culture); p=0.02 (LAMPS)]. ANOVA analysis across all clones treated with E2 produced significant p-values for monoculture (p=0.02), co-culture (p=0.02), and LAMPS (p=0.03. One-way ANOVA analysis comparing WT clones treated with E2 compared to the constitutive growth of ESR1 mutants did not result in significant differences under any culture condition. These results also demonstrate a phenotypic switch in estrogen-dependent growth that depends upon TME composition, indicating that these phenotypes are not a result from variation among different clonal cell lines. In both clone sets, in addition to significant constitutive growth (red asterisks), both D538G and Y537S mutations confer enhanced estrogen-dependent growth that is sensitive to the composition of the TME. While D538G clones show enhanced estrogen-dependent growth exclusively in 2D monoculture (A), Y537S-expressing cells display this growth advantage only in co-culture and LAMPS (B, C).

Figure S5. Comparison of the effects of Zone 1 and Zone 3 oxygen tensions on albumin secretion in LAMPS and co-culture models. Albumin secretion was

measured by ELISA in the efflux media at the days indicated in both LAMPS (A-C) and co-culture models (D-F) as previously described [2,4]. Results are shown as the mean ± SD from 3 LAMPS devices or plate wells. Albumin output of LAMPS devices maintained at zone 1 (12-15%) was higher than that observed in LAMPS models maintained at zone 3 (3-6%) oxygen tensions. For co-culture models, a similar trend is observed for plates maintained at zone 1 (20%) and zone 3 (5%) oxygen tensions, but the overall amount of albumin output is lower than in LAMPS devices, consistent with previous work [2,4].

Figure S6. Neither WT nor ESR1 mutant-expressing cells confer resistance to the chemotherapeutic agent doxorubicin. WT (blue bars), Y537S (red bars), and D538G (green bars) expressing cells were grown in the presence of 5 nM E2 in both 2D monoculture and static co-culture. Cells were maintained in either zone 1 (20%) or zone 3 (5%) oxygen tension and were treated over a 13-day time course with the chemotherapeutic agent doxorubicin at 1μM or 0. 1μM [7]. Graphs are displayed as the percent of vehicle treated control for each ESR1 expressing cell line where the mean change in fluorescence intensity ± SD of three individual fields was quantified. P-values were obtained by using an unpaired, 2-tailed t-test (* = $p < 0.05$) to compare the growth of WT and mutant expressing cells in the presence of doxorubicin. Neither WT nor ESR1 mutant expressing cells demonstrated resistance to either concentration of doxorubicin tested.

Figure- S1

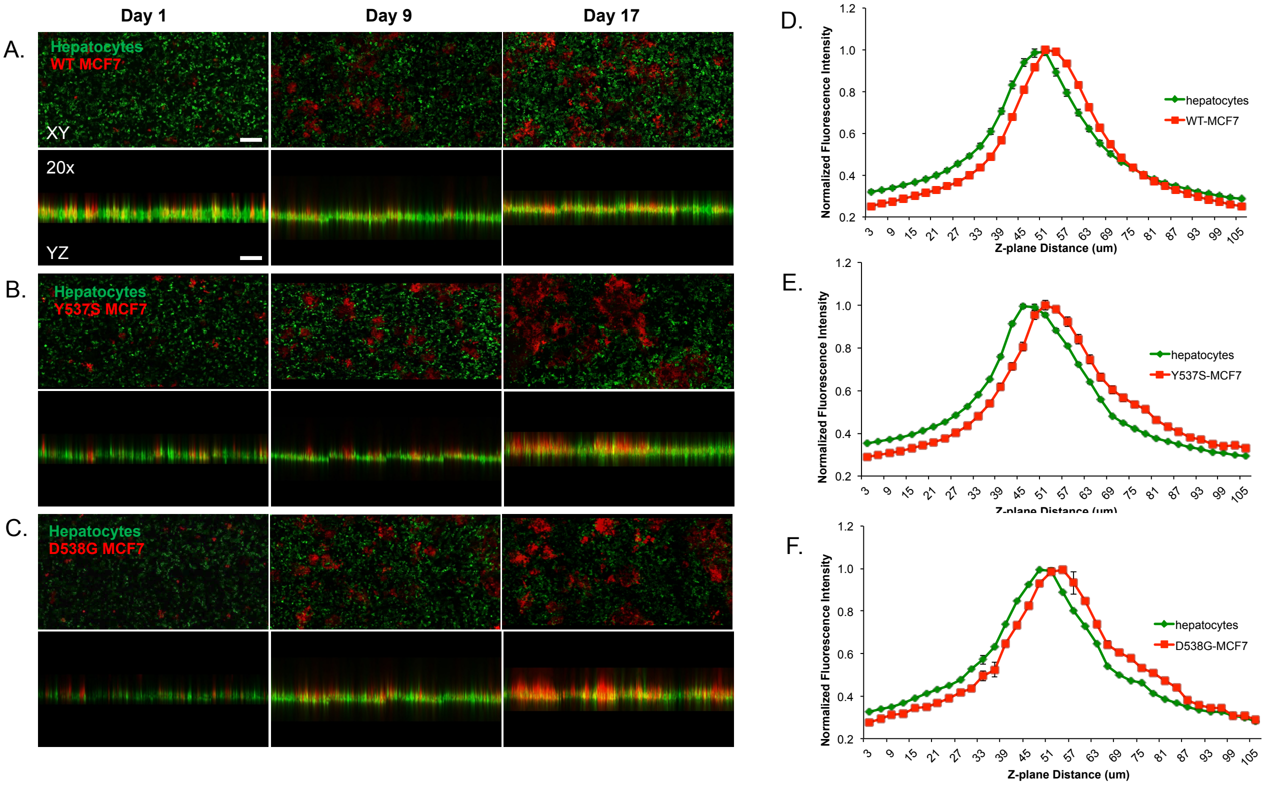
Day 1
Day 9
Day 17
A.
Hepatocytes
WT MCF7
XY
20x
YZ
B.
Hepatocytes
Y537S MCF7
C.
Hepatocytes
D538G MCF7
D.
E.
F.
Normalized Fluorescence Intensity
Z-plane Distance (um)
hepatocytes
WT-MCF7
Y537S-MCF7
D538G-MCF7

Figure- S2

A.

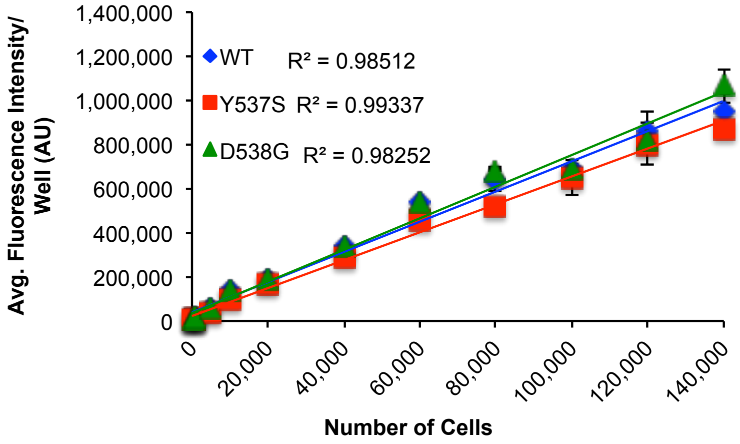

B.

	Avg Fluorescence Intensity (AU)		
**# of Cells**	**WT**	**Y537S**	**D538G**
**200**	4,016 ± 697	3,573 ± 363	4,097 ± 229
**500**	8,220 ± 593	7,149 ± 549	9,151 ± 1,115
**1,000**	14,973 ± 632	10,668 ± 1,162	15,474 ± 201
**5,000**	54,864 ± 7,700	41,412 ± 1,762	58,947 ± 8,410
**10,000**	138,094 ± 4,820	98,689 ± 7,130	139,711 ± 1,092
**20,000**	186,787 ± 6,912	170,218 ± 3,722	189,672 ± 1,775
**40,000**	340,705 ± 1,730	284,730 ± 4,291	341,407 ± 4,022
**60,000**	536,115 ± 6,234	460,637 ± 26,578	541,647 ± 2,267
**80,000**	643,989 ± 58,655	521,735 ± 18,672	682,955 ± 6,620
**100,000**	689,017 ± 21,088	649,930 ± 79,004	691,689 ± 18,311
**120,000**	855,694 ± 90,767	803,432 ± 98,189	818,470 ± 32,801
**140,000**	947,085 ± 85,141	867,175 ± 7,283	1,065,238 ± 78,968

Figure- S3

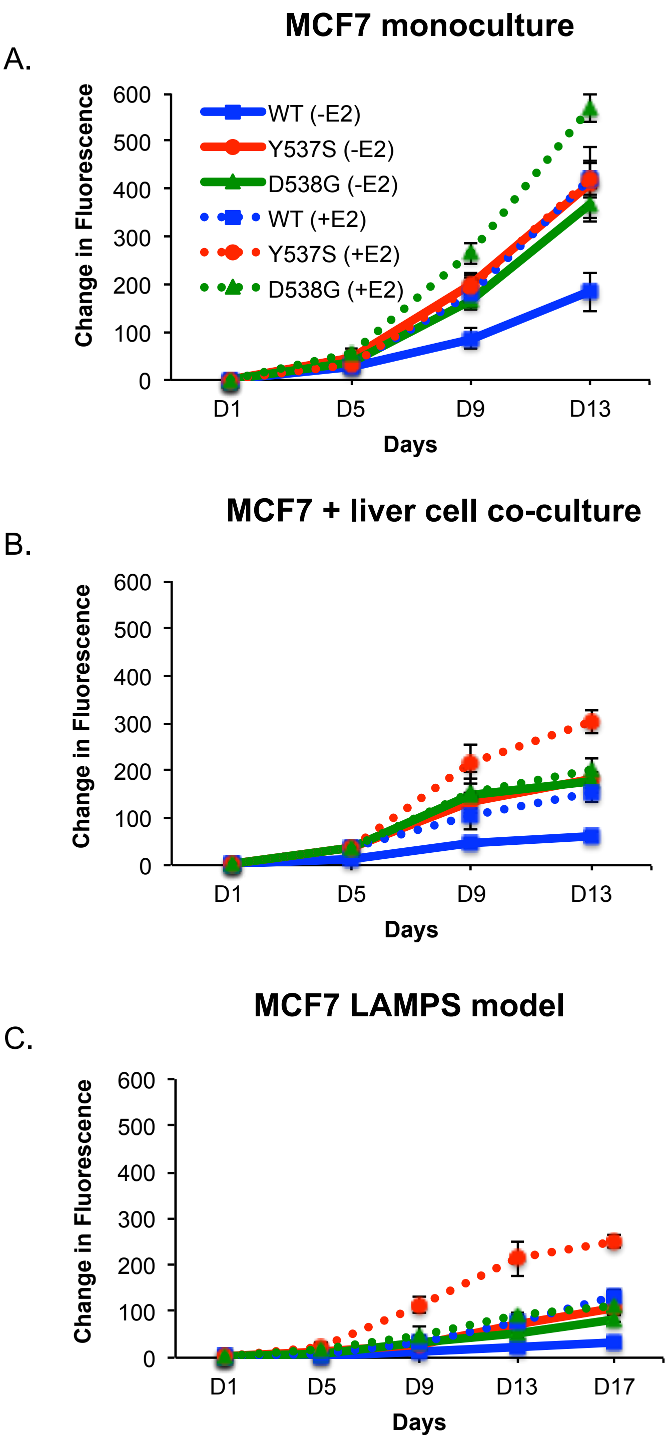
MCF7 monoculture
A.
Change in Fluorescence
600
500
400
300
200
100
0
WT (-E2)
Y537S (-E2)
D538G (-E2)
WT (+E2)
Y537S (+E2)
D538G (+E2)
D1
D5
D9
D13
Days
MCF7 + liver cell co-culture
B.
Change in Fluorescence
600
500
400
300
200
100
0
D1
D5
D9
D13
Days
MCF7 LAMPS model
C.
Change in Fluorescence
600
500
400
300
200
100
0
D1
D5
D9
D13
D17
Days

Figure- S4

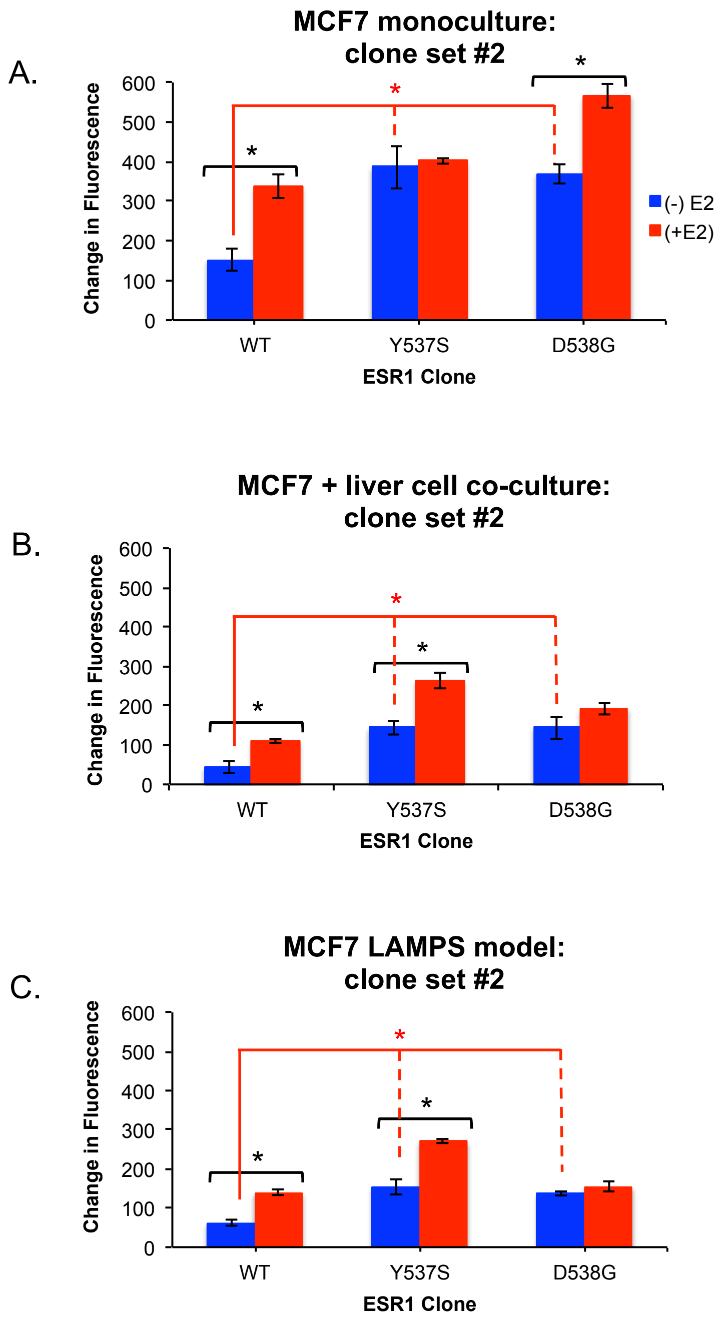
A.
MCF7 monoculture:
clone set #2
B.
MCF7 + liver cell co-culture:
clone set #2
C.
MCF7 LAMPS model:
clone set #2
Change in Fluorescence
ESR1 Clone
WT
Y537S
D538G
(-) E2
(+E2)
0
100
200
300
400
500
600
*

Figure- S5

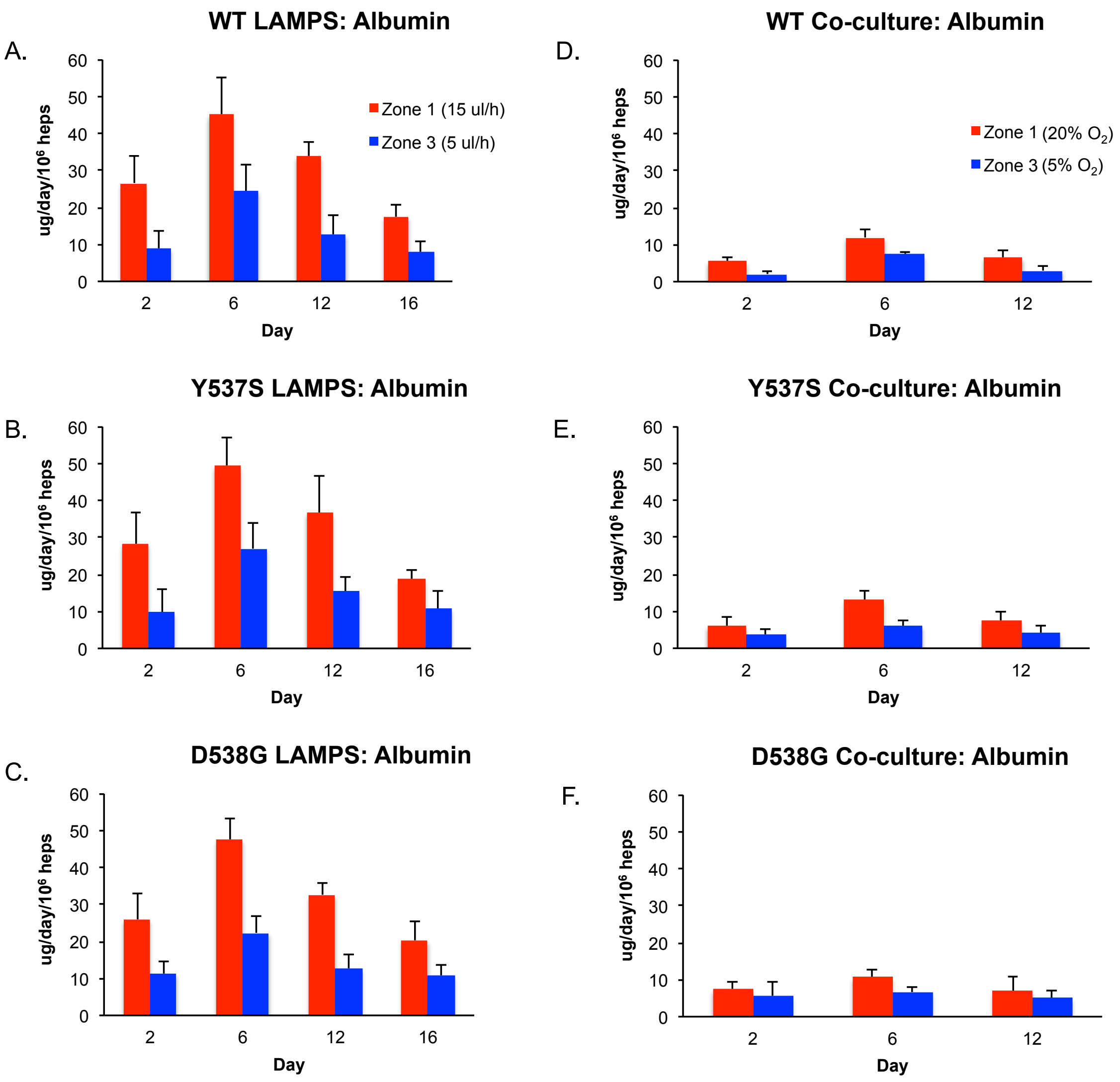
A.
WT LAMPS: Albumin
ug/day/10⁶ heps
Zone 1 (15 ul/h)
Zone 3 (5 ul/h)
Day
B.
Y537S LAMPS: Albumin
C.
D538G LAMPS: Albumin
D.
WT Co-culture: Albumin
Zone 1 (20% O2)
Zone 3 (5% O2)
E.
Y537S Co-culture: Albumin
F.
D538G Co-culture: Albumin

Figure- S6

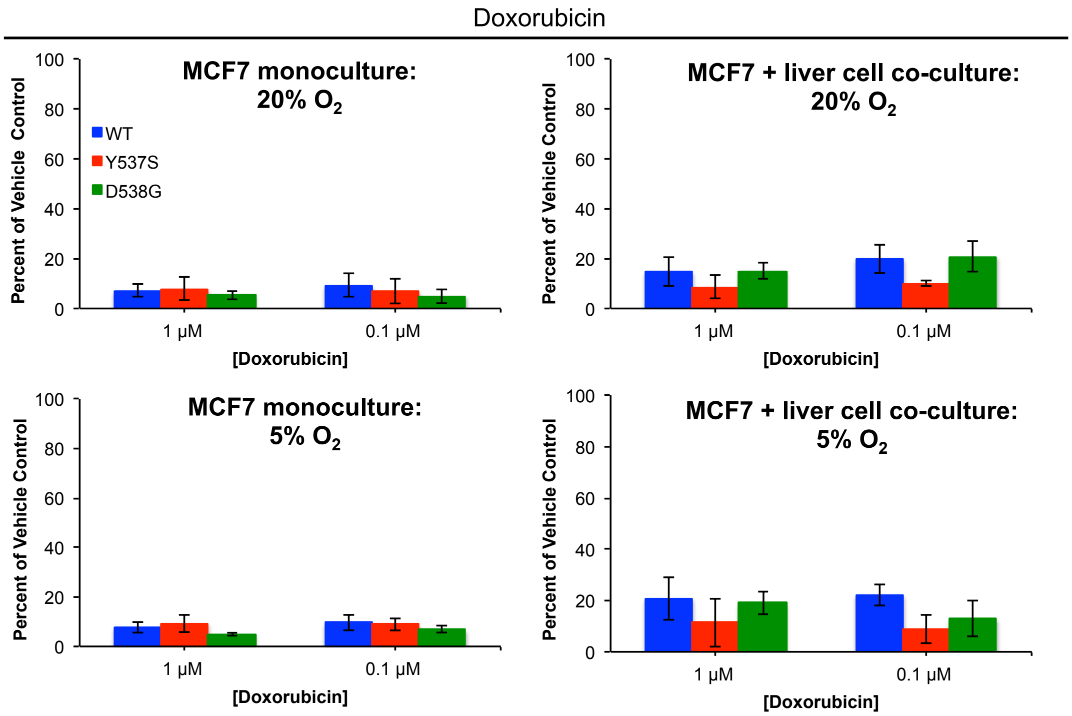
Doxorubicin
MCF7 monoculture: 20% O_2
MCF7 + liver cell co-culture: 20% O_2
MCF7 monoculture: 5% O_2
MCF7 + liver cell co-culture: 5% O_2
WT
Y537S
D538G
Percent of Vehicle Control
[Doxorubicin]
1 μM
0.1 μM
0
20
40
60
80
100

Supplemental Tables

Table S1. Cell Seeding Densities in LAMPS and Co-culture Models.

Model	Cell Type	Seeding Number	% of Total Cells
**LAMPS**	hepatocytes	275,000	50%
	endothelial	150,000	30%
	Kupffer	80,000	16%
	stellate	20,000	4%
	MCF7	300	0.1%
**Co-culture**	hepatocytes	50,000	50%
	endothelial	30,000	30%
	Kupffer	16,000	16%
	stellate	4,000	4%
	MCF7	300	0.3%

Table S2. Mass Spectrometry Analysis of LAMPS model efflux fluid.

Compound (carrier protein)	% of Drug Recovery in efflux media	
	- Carrier Protein	+ Carrier Protein
**β-Estradiol (SHBG)**	83	87
**Fulvestrant (LDL)**	6	56
**AZD9496 (LDL)**	95	95
**Doxorubicin (LDL)**	30	27

To assess the drug binding capability of the polydimethylsiloxane (PDMS)-containing LAMPS device for compounds used in these studies (β-estradiol, fulvestrant, AZD9496, and doxorubicin), we used perfusion flow tests and mass spectrometry analysis of efflux to determine the overall effective concentration of each compound as previously described [5]. Cell-free LAMPS devices were coated with collagen (200 μg/mL)/fibronectin (100 μg/mL) and washed with PBS prior to incubation with hepatocyte maintenance media containing either 5 nM estradiol or 2 μM drug (fulvestrant, AZD9496, or doxorubicin) -/+ the indicated carrier protein at 0.1 mg/mL. For estradiol, sex hormone binding globulin (SHBG) was used as a carrier while low-density lipoprotein (LDL) was used as a carrier for fulvestrant, AZD9496, and doxorubicin. LAMPS devices were incubated for 72 h and the amount of estradiol or drug present in the efflux media compared to the initial input was determined by mass spectrometry and expressed as a percentage of the starting input media in the presence or absence of carrier protein.
